# 6-OHDA generated ROS induces DNA damage and p53- and PUMA-dependent cell death

**DOI:** 10.1186/1750-1326-6-2

**Published:** 2011-01-06

**Authors:** Alison I Bernstein, Sean P Garrison, Gerard P Zambetti, Karen L O'Malley

**Affiliations:** 1 Department of Anatomy and Neurobiology, Washington University School of Medicine, St. Louis, MO 63110, USA; 2 Department of Biochemistry, St. Jude Children's Research Hospital, Memphis, TN 38105, USA

## Abstract

**Background:**

Parkinson's disease (PD) is characterized by the selective loss of dopaminergic neurons in the substantia nigra (SN), resulting in tremor, rigidity, and bradykinesia. Although the etiology is unknown, insight into the disease process comes from the dopamine (DA) derivative, 6-hydroxydopamine (6-OHDA), which produces PD-like symptoms. Studies show that 6-OHDA activates stress pathways, such as the unfolded protein response (UPR), triggers mitochondrial release of cytochrome-c, and activates caspases, such as caspase-3. Because the BH3-only protein, Puma (p53-upregulated mediator of apoptosis), is activated in response to UPR, it is thought to be a link between cell stress and apoptosis.

**Results:**

To test the hypothesis that Puma serves such a role in 6-OHDA-mediated cell death, we compared the response of dopaminergic neurons from wild-type and *Puma*-null mice to 6-OHDA. Results indicate that Puma is required for 6-OHDA-induced cell death, in primary dissociated midbrain cultures as well as *in vivo*. In these cultures, 6-OHDA-induced DNA damage and p53 were required for 6-OHDA-induced cell death. In contrast, while 6-OHDA led to upregulation of UPR markers, loss of ATF3 did not protect against 6-OHDA.

**Conclusions:**

Together, our results indicate that 6-OHDA-induced upregulation of *Puma *and cell death are independent of UPR. Instead, p53 and DNA damage repair pathways mediate 6-OHDA-induced toxicity.

## Background

PD is a common neurodegenerative disorder characterized by the progressive loss of dopaminergic neurons in the SN resulting in the loss of dopaminergic innervation to the striatum. Although the molecular mechanisms are unknown, oxidative stress, mitochondrial dysfunction and endoplasmic reticulum (ER) stress have all been implicated in the etiology of this disorder [1,2].

The neurotoxin 6-OHDA is a hydroxylated analog of DA that is commonly used to model dopaminergic degeneration both *in vitro *and *in vivo *[3]. Like DA, 6-OHDA quickly oxidizes to form a variety of free radical species, including hydrogen peroxide, superoxide and hydroxyl radicals [4]. Toxin-induced free radical formation can be blocked by antioxidants, such as N-acetyl-L-cysteine (NAC), MnTBAP, or C3 carboxyfullerene [5,6], which prevent downstream toxic sequelae such as oxidation of proteins [7-9] and cell death [10-12]. 6-OHDA-oxidized proteins cause ER stress and upregulation of the UPR [7,13-15], which regulates protein folding, protein degradation and protein translation.

In addition to UPR, 6-OHDA also induces ROS-dependent apoptosis in dopaminergic cells [6,7]. Apoptosis is regulated by the Bcl-2 family of proteins in which BH3-only proteins are recognized as essential initiators of this process. In particular, the BH3-only protein, Puma, is activated in response to a variety of death stimuli, including DNA damage, ER stress, and oncogene-mediated hyperproliferation [16-18]. Each of these insults induces PUMA expression, resulting in cytochrome-c release from the mitochondria, caspase activation and apoptosis [17,19,20]. In addition, cells deficient in Puma are resistant to ER stress- and DNA damage-induced apoptosis [16,18]. These data are consistent with studies suggesting that when UPR pathways are overwhelmed apoptosis is triggered [21,22]. Thus, Puma may provide a link between ER stress, UPR and apoptosis.

Since 6-OHDA triggers these same pathways, we proposed that Puma may mediate 6-OHDA toxicity [16-18]. Here, we show that Puma is required for 6-OHDA-induced apoptosis in primary dissociated midbrain cultures and *in vivo*. Using animals deficient in key DNA damage and UPR pathway components, we also show that toxin-mediated apoptosis is independent of the upregulation of UPR. While UPR may be protective, the activation of the DNA damage pathway plays a more direct and essential role in mediating apoptosis in this PD model.

## Results

### 6-OHDA upregulates Puma

**Figure 1:**
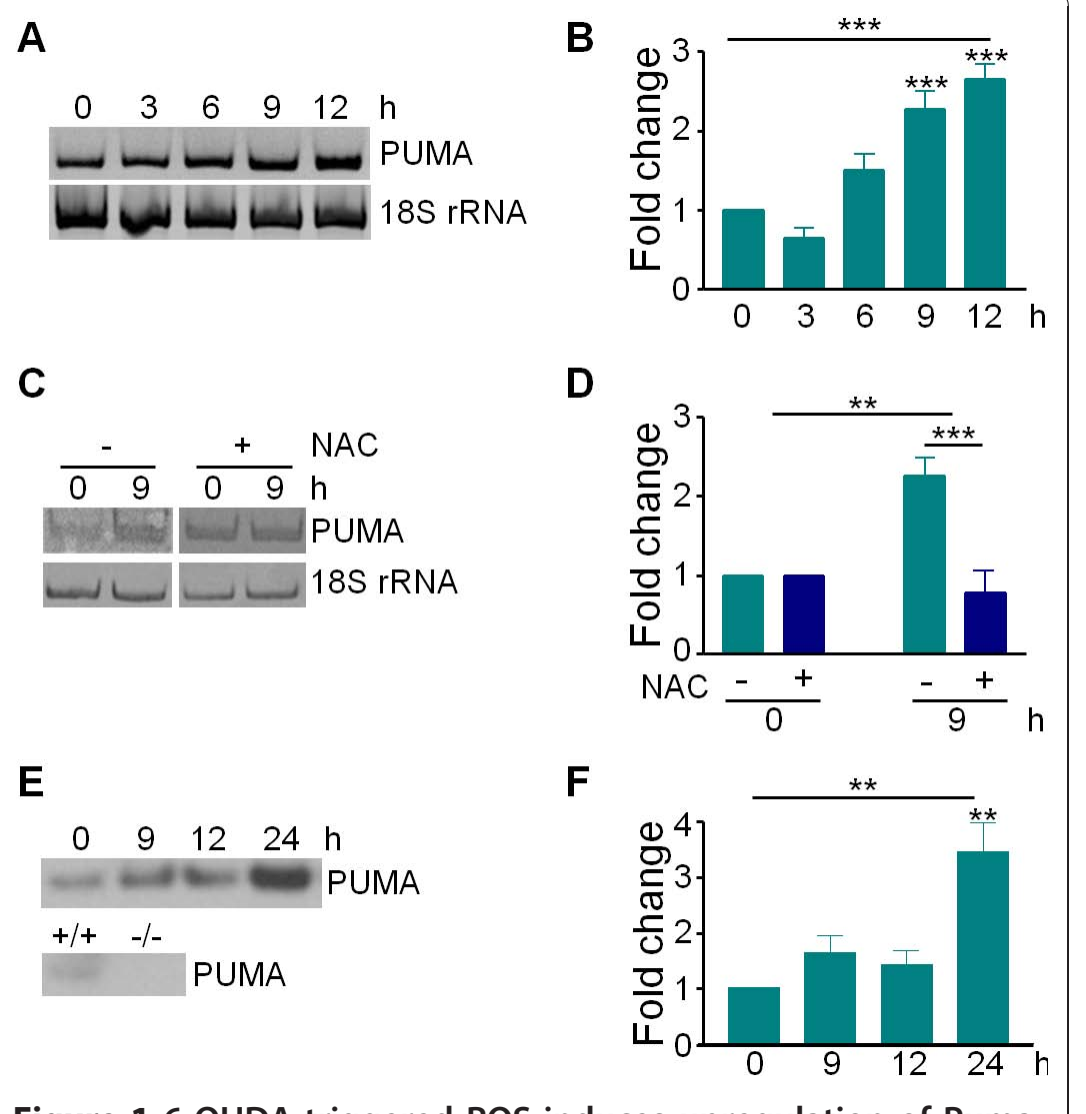
**6-OHDA-triggered ROS induces upregulation of Puma**. Dissociated dopaminergic cultures derived from C57Bl/6 mice were treated with 20 ¿M 6-OHDA and total RNA was collected at the indicated times. A) Levels of *Puma *and 18S rRNA were analyzed by RT-PCR. B) Gels in A were quantitated in ImageQuant and analyzed by one-way ANOVA (***, p < 0.001) with Bonferroni post-tests to compare each time point to untreated (9 hr and 12 hr: ***, p < 0.001). C) Cultures were treated with 5 mM NAC immediately prior to treatment with 20 ¿M 6-OHDA. Total RNA was collected and analyzed as in A. D) Gels in A were quantitated in ImageQuant and analyzed by two-way ANOVA (6-OHDA treatment: **, p < 0.01; NAC pre-treatment: ***, p < 0.001). E) Cell lysates were collected in RIPA buffer at the indicated times after treatment with 20 ¿M 6-OHDA. Protein levels were analyzed by western blotting for Puma. Antibody specificity was confirmed by comparing blots of known tissues positive and negative for Puma. F) Western blots in C were quantitated in ImageQuant. Levels of Puma were normalized to actin levels. Data were analyzed by one-way ANOVA (**, p < 0.01) with Bonferroni post-tests to compare each time point to untreated (24 hr: **, p < 0.01).

Previous results from our lab demonstrated that 6-OHDA causes an increase in ROS and an ROS-dependent upregulation of UPR and apoptosis. Temporally, UPR was rapidly induced within 1-3 hours, preceding mitochondrial induction of apoptosis by 12-15 hours [7,17]. Since PUMA has been demonstrated to be induced by ER stress and to trigger mitochondrial events relating to apoptosis, we sought to determine if PUMA is transcriptionally upregulated in response to 6-OHDA. Therefore, dissociated dopaminergic cultures were treated with 20 ¿M 6-OHDA, a dose that produces 50% loss after 24 hours, and analyzed by RT-PCR and western blot. Levels of *Puma *mRNA were significantly increased by 9 hours after treatment with 6-OHDA (Figure 1A, B). No increase in *Puma *RNA was seen in cultures pre-treated with the anti-oxidant NAC, which we have previously demonstrated blocks 6-OHDA-induced ROS, UPR and apoptosis [7] (Figure 1C, D). Surprisingly, even though *Puma *mRNA was upregulated by 9 hours after 6-OHDA treatment, Puma protein did not significantly increase until 24 hours (Figure 1E, F). Although the source of this discrepancy is unclear, it is possible that western blot sensitivity was sufficiently variable as to prevent detection of smaller changes. These results demonstrate that 6-OHDA induces the upregulation of Puma in a ROS-dependent manner, suggesting that Puma plays a role in 6-OHDA-mediated cell death.

### Loss of Puma protects primary dopaminergic and non-dopaminergic neurons against 6-OHDA

**Figure 2:**
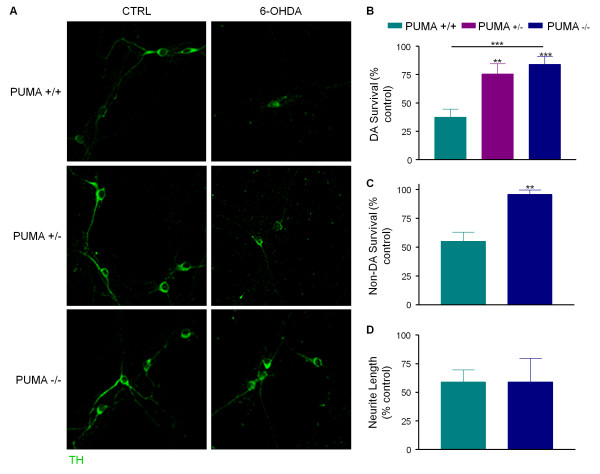
**Puma is required for 6-OHDA-induced cell death**. Dissociated dopaminergic cultures derived from individual animals from *Puma *+/- × *Puma *+/- matings were treated with 20 ¿M 6-OHDA for 24 hours. A) Cells were fixed and stained for TH. B) TH-positive cells were counted by an unbiased stereological method and survival expressed as a percentage of untreated control. Data were analyzed by one-way ANOVA (***, p < 0.001) with Bonferroni post-tests to compare to wild-type (+/-: **, p < 0.01; -/-: ***, p < 0.001) C) Cultures co-stained for TH and NeuN were counted to assess the survival of non-DA neurons. Data were analyzed by Student's t-test (**, p < 0.01) D) TH-positive neurite length was estimated by an unbiased stereological method. Data were analyzed by Student's t-test (ns).

To determine if Puma plays an essential role in 6-OHDA toxicity, dissociated dopaminergic cultures from *Puma *+/+, +/- and -/- littermates were treated with or without drug. After 24 hours, cultures were stained for tyrosine hydroxylase (TH), a marker of dopaminergic neurons; counting TH-positive cells showed that loss of Puma significantly protected these neurons from cell death (Figure 2A, B). Counting of cultures co-stained with TH and NeuN demonstrated that Puma-deficiency also rescued non-dopaminergic neurons (Figure 2C). This is consistent with previous findings showing 6-OHDA-induced cell death is not as specific to dopaminergic neurons *in vitro *as it is *in vivo *[23]. Because many recent studies show that neurite degeneration is an active process distinct from that of cell death [24], loss of neurites was also assessed. In contrast to the survival of *Puma *-/- cell bodies, there was no protection of *Puma *-/- neurites (Figure 2A, D). These data indicate that Puma mediates 6-OHDA toxicity in cell bodies, but not in neurites.

### Puma deficiency protects dopaminergic neurons *in vivo *against 6-OHDA

**Figure 3:**
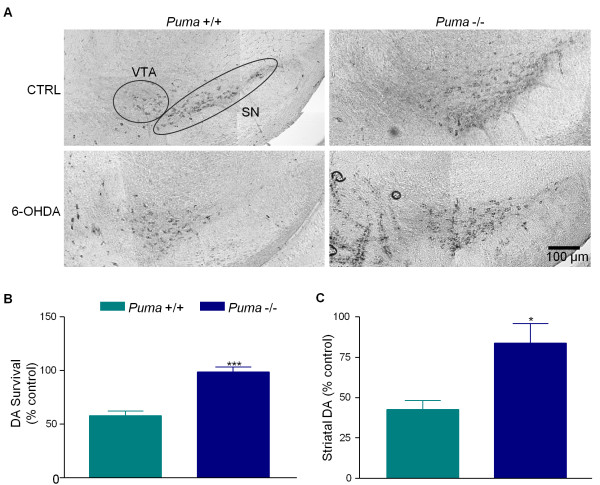
**Puma is required for 6-OHDA-induced loss of DA neurons in the SN and striatal DA depletion**. C57 and *Puma *-/- mice were treated with 6-OHDA by unilateral intrastriatal injection and sacrificed one month later. A) 50 ¿M sections were stained for TH. Pictures are representative images of the SN ipsilateral to injection site. Locations of SN and ventral tegmental area (VTA) are indicated in the first panel. B) TH-positive cells were counted, ipsilateral to contralateral ratios were calculated and survival was expressed as a percentage of untreated control. Data were analyzed by two-way ANOVA (treatment: **, p < 0.01; genotype: **, p < 0.01). Results of Bonferroni post-tests are indicated on graph (***, p < 0.001). C) Striata were lysed and DA levels were analyzed by HPLC with electrochemical detection. Ipsilateral to contralateral ratios were calculated and expressed as a fraction of untreated control. Data were analyzed by two-way ANOVA (treatment: **, p < 0.01; genotype: ns). Results of Bonferroni post-tests are indicated on graph (*, p < 0.05).

Since loss of PUMA blocked 6-OHDA-induced cell death in cultured dopaminergic neurons, we tested whether loss of PUMA is protective *in vivo*. Following a preliminary dose response experiment (data not shown), unilateral intrastriatal injections of 6-OHDA or saline were done with wild-type and *Puma *-/- mice. One month later animals were sacrificed and surviving TH-positive neurons in the SN were counted by an unbiased stereological method to assess dopaminergic cell survival [25]. Unbiased stereology has been widely used to assess neuronal number in many brain regions, including TH-positive neurons in the SN [26]. In wild-type mice, 50% of TH-positive neurons in the SN were lost while TH-positive neurons in the ventral tegmental area were unaffected. Although most dopaminergic cells would have been destroyed within a week after injection, animals were assessed at one month to ensure stability of response and to allow for behavioral measurements. Viability and variability proved to be problematic in mice, however, disallowing meaningful behavioral assessments. Loss of Puma significantly rescued dopaminergic neurons in the SN, indicating that Puma is critical for 6-OHDA-induced cell loss *in vivo *as well as *in vitro *(Figure 3A, B). Puma-deficiency also prevented the loss of dopamine in the striata of 6-OHDA-injfinected brains, suggesting that Puma loss also prevented dopaminergic terminal field destruction (Figure 3C).

### Puma-deficiency prevents 6-OHDA-induced apoptosis

**Figure 4:**
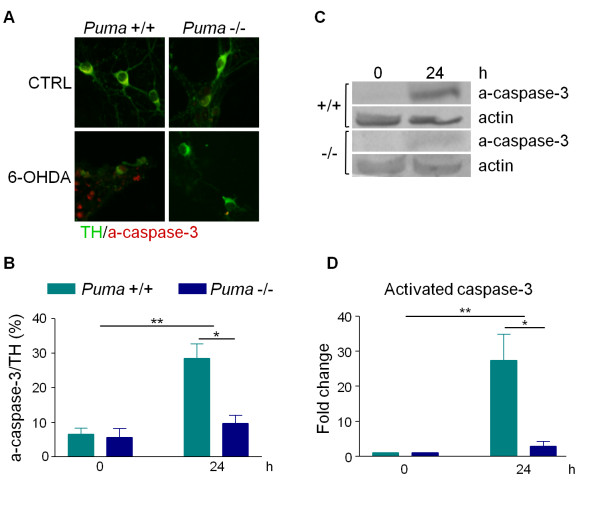
**Puma mediates caspase-3 activation following 6-OHDA treatment**. Dopaminergic cultures derived from *Puma *+/+ and *Puma *-/- animals were treated with 20 ¿M 6-OHDA. A) Cultures were fixed and stained at 0 or 24 hours for TH and cleaved caspase-3 (a-caspase-3). B) TH-positive and cleaved caspase-3-positive cells were counted and the percentage of TH-positive cells that were also cleaved caspase-3 positive was calculated. Data were analyzed by two-way ANOVA (treatment: **, p < 0.01; genotype: *, p < 0.05). C) Cell lysates were collected in RIPA buffer 24 hours after treatment with 20 ¿M 6-OHDA. Protein levels were analyzed by western blotting for cleaved caspase-3 and actin. D) Western blots in C were quantitated in ImageQuant. Levels of cleaved caspase-3 were normalized to actin levels. Data were analyzed by two-way ANOVA (treatment: **, p < 0.01; genotype: *, p < 0.05).

Previously, we have shown that 6-OHDA-generated ROS induces caspase-3 activation 12-15 hours post treatment [7,27]. Since PUMA is known to induce apoptosis via activation of caspases-3 in other systems, we tested by immunocytochemistry and western blotting whether loss of Puma prevents caspase-3 cleavage after 6-OHDA treatment. Immunofluorescent analysis followed by unbiased counting of cultures prepared from either *Puma *+/+ or -/- animals revealed that the percentage of TH neurons that were positive for cleaved caspase-3 prior to toxin treatment was unchanged in *Puma *-/- cultures (Figure 4A, B), while cleaved caspase-3 positive neurons increased threefold in *Puma *+/+ cultures (Figure 4A, B). Western blotting confirmed that *Puma *+/+ cultures exhibited a large increase in the levels of cleaved caspase-3 at 24 hours, while *Puma *-/- cultures did not (Figure 4C, D). These results indicate that Puma is required for the activation of caspase-3 in dopaminergic neurons in response to 6-OHDA.

### Activation of UPR Markers is unaffected by Puma-deficiency

**Figure 5:**
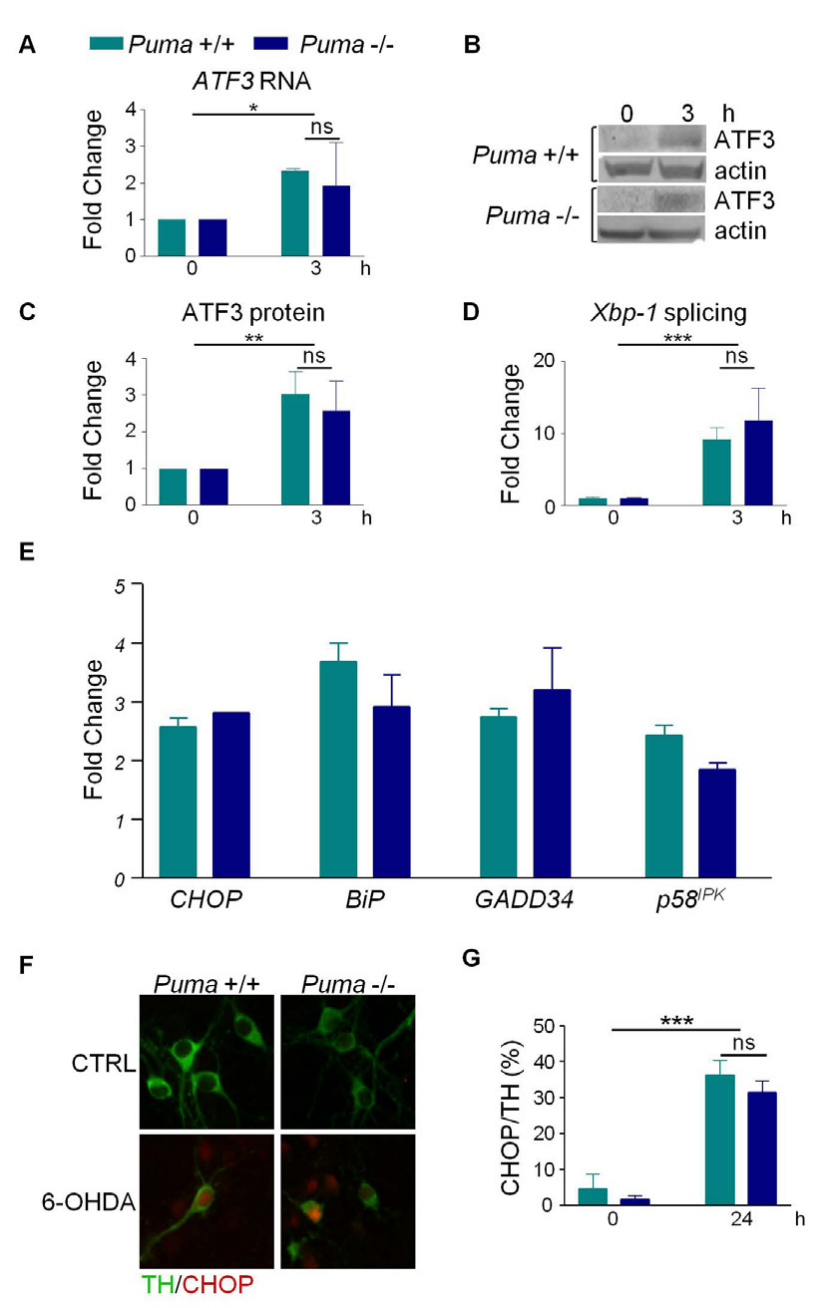
**Loss of Puma does not alter the activation of UPR markers**. Dopaminergic cultures derived from *Puma *+/+ and -/- animals were treated with 20 ¿M 6-OHDA. A) Total RNA was isolated at the indicated times and *ATF3 *and *GAPDH *RNA levels were analyzed by qPCR. *ATF3 *levels were normalized to *GAPDH *levels. B) Cell lysates were collected in RIPA buffer at the indicated times and protein levels were analyzed by western blotting for ATF3 and actin. C) Western blots in B were quantitated in ImageQuant. Levels of ATF3 were normalized to actin levels. Data were analyzed by two-way ANOVA (treatment: **, p < 0.01; genotype: ns). D) Total RNA was isolated at the indicated times and *Xbp-1 *RNA levels were analyzed by semi-quantitative RT-PCR. Data were analyzed by two-way ANOVA (treatment: ***, p < 0.001; genotype: ns). E) Total RNA was isolated 9 hours after treatment and RNA levels of the indicated genes were analyzed by qPCR. Data are shown as fold change over untreated control and were analyzed by two-way ANOVA. *CHOP *(treatment: ***, p < 0.001; genotype: ns), *BiP *(treatment***, p < 0.001; genotype: ns), *GADD34 *(treatment: **, p < 0.01; genotype: ns), *p58IPK *(treatment: **, p < 0.01; genotype: ns). F) Cells were fixed 24 hours after treatment and stained for TH and CHOP. G) TH-positive and CHOP-positive cells were counted and the percentage of TH-positive cells that were also CHOP-positive was calculated. Data were analyzed by two-way ANOVA (treatment: ***, p < 0.001; genotype: ns).

If Puma serves as a link between UPR and the mitochondrial induction of apoptosis, then early events of UPR, such as ATF3 induction and Xbp-1 splicing, should not be affected by Puma-deficiency. Accordingly, early UPR events will be upregulated by 6-OHDA in both *Puma *+/+ and *Puma *-/- cultures. As predicted, ATF3 RNA and protein levels were increased to a similar extent in both *Puma *+/+ and -/- cultures 3 hours after treatment with 6-OHDA (Figure 5A-C). Puma-deficiency also had no effect on 6-OHDA-mediated induction of Xbp-1 splicing or the overall levels of Xbp-1 (Figure 5D, data not shown). Consistent with the proposed model, these early UPR markers are induced by 6-OHDA regardless of *Puma *status indicating that they are not downstream of Puma.

The timing of PUMA upregulation suggests that it is either upstream of or parallel to the induction of late UPR markers. Therefore, we assessed the expression of late UPR markers in *Puma *+/+ and -/- cultures 9 hours after 6-OHDA treatment, a time point at which these markers are significantly upregulated [7,27]. RNA prepared from either PUMA wild type or null dopaminergic cultures showed that markers representing all 3 branches of the UPR pathway (*CHOP, BiP, GADD34*, and *p58^IPK^*) were upregulated to a similar extent in response to toxin regardless of genotype (Figure 5E). Consistent with the qPCR results, the UPR marker CHOP was increased in the nuclei of dopaminergic neurons following toxin treatment in both *Puma *+/+ and -/- cultures (Figure 5F, G). Collectively, these data suggest that loss of Puma does not significantly affect the UPR pathway.

### Loss of ATF3 does not protect cells against 6-OHDA

**Figure 6:**
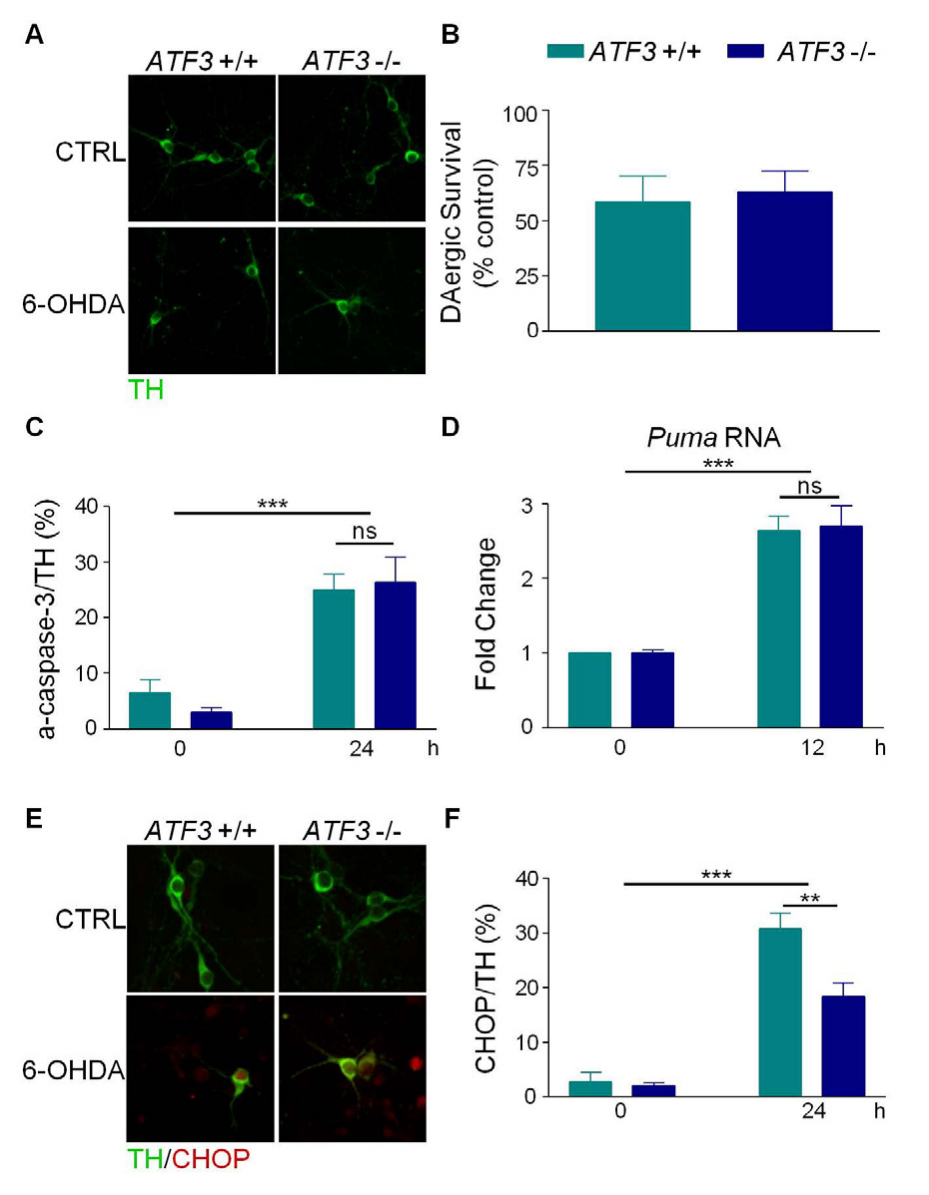
**ATF3 is not required for 6-OHDA-induced cell death**. Dopaminergic cultures prepared from *ATF3 *+/+ and -/- mice were treated with 20 ¿M 6-OHDA. A) 24 hours after treatment, cultures were fixed and stained for TH. B) TH-positive cells were counted and survival expressed as a percent of untreated control. Data were analyzed by Student's t-test (ns). C) Twenty four hours after treatment, cells were fixed and stained for TH and cleaved caspase-3 (a-caspase-3). TH-positive and a-caspase-3 positive cells were counted and the percentage of TH-positive cells that were also a-caspase-3-positive was calculated. Data were analyzed by two-way ANOVA (treatment: ***, p < 0.001; genotype: ns). D) Total RNA was isolated at the indicated times and *Puma *and *18S rRNA *RNA levels were assayed by RT-PCR. Data were analyzed by two-way ANOVA (treatment: ***, p < 0.001, genotype: ns). E) Cells were fixed 24 hours after treatment and stained for TH and CHOP. F) TH-positive and CHOP-positive cells were counted and the percentage of TH-positive cells that were also CHOP-positive was calculated. Data were analyzed by two-way ANOVA (treatment: ***, p < 0.001; genotype: *, p < 0.05).

The transcription factor ATF3 is rapidly induced in response to ER stress and is a key mediator of the PERK branch of the UPR pathway [28]. Since ATF3 levels increase dramatically in response to 6-OHDA (> 30-fold) [7,27] and the levels of various UPR markers are unaffected by loss of *Puma*, we sought to determine if 6-OHDA-mediated UPR was playing a direct role in the death of these neurons using *ATF3*-deficient mice [29]. Dissociated dopaminergic cultures from *ATF3 *+/+ and -/- mice were treated with or without 6-OHDA for 24 hours before being evaluated for surviving TH-positive cells. Loss of *ATF3 *did not protect cells or neurites against 6-OHDA (Figure 6A, B, data not shown). We also found that 6-OHDA-induced caspase-3 cleavage in dopaminergic neurons regardless of the *ATF3 *genotype (Figure 6C). These results indicate that ATF3 is not required for cell death induced by 6-OHDA and suggest that UPR does not play a direct role in the induction of apoptosis by 6-OHDA.

In addition, we tested whether *Puma *expression levels were altered in the *ATF3*-deficiency model. Consistent with the sensitivity of *ATF3 *-/- dopaminergic neurons to 6-OHDA, *Puma *was upregulated to the same extent in both wild-type and *ATF3*-deficient cultures in response to neurotoxin treatment (Figure 6D). Because CHOP is upregulated by all three branches of the UPR pathway including the PERK/ATF3 branch [21], we directly assessed CHOP levels in dopaminergic neurons derived from *ATF3 *+/+ or *ATF3 -/- *animals. CHOP was still induced in the *ATF3*-deficient animals, albeit to a lesser extent (Figure 6E, F). That this was only a partial reduction is not surprising since IRE1 and ATF6 also induce CHOP [30]. These data indicate that ATF3 deficiency does not affect 6-OHDA-induced cell loss even though it attenuates CHOP upregulation, suggesting that prolonged 6-OHDA-mediated CHOP upregulation does not result in apoptosis.

### 6-OHDA leads to DNA damage and activation of p53

**Figure 7:**
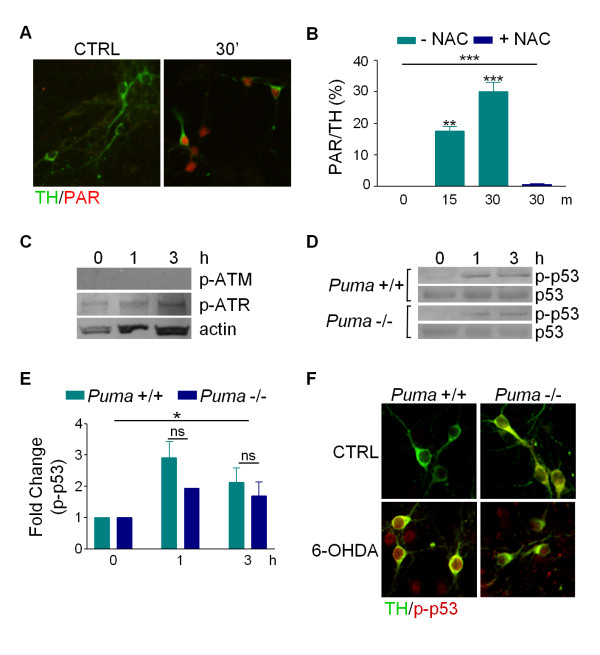
**6-OHDA leads to activation of the DNA damage repair pathway**. Primary cultures prepared from *Puma *+/+ and -/- mice were treated with 20 ¿M 6-OHDA. A) Cultures were fixed 30 minutes after treatment and stained for TH and PAR. B) Cultures treated for 15 minutes, 30 minutes, or 30 minutes with 5 mM NAC pretreatment were fixed and stained for TH and PAR. TH-positive and PAR-positive cells were counted and the percentage of TH-positive cells that were also PAR-positive was calculated. Data were analyzed by one-way ANOVA (***, p < 0.001) with Bonferroni post-tests to compare each treatment to untreated (15 m: **, p < 0.01; 30 m: ***, p < 0.001). C and D) Cell lysates were collected in RIPA buffer at the indicated times and proteins levels were assessed by western blotting for p-Atm, p-Atr, p-p53, p53 and actin. E) Western blots in D were quantitated with ImageQuant. Levels of p-p53 were normalized to total p53 levels. Data were analyzed by two-way ANOVA (treatment: *, p < 0.05; genotype: ns). F) Cultures treated for 1 hour with 6-OHDA were fixed and stained for TH and p-p53.

Since activation of UPR does not appear to mediate cell death in response to 6-OHDA, we sought to determine how PUMA is upregulated in this model. Since ROS can induce DNA damage in addition to protein damage, we determined if 6-OHDA-generated ROS induces the latter by assessing levels of poly (ADP-ribose) (PAR) in dissociated dopaminergic cultures. PAR is synthesized by the nuclear DNA repair enzyme PAR polymerase in response to DNA strand breaks [31,32]. Within 15 minutes of 6-OHDA treatment, PAR staining in dopaminergic neurons was dramatically increased and was blocked by NAC (Figure 7A, B). These results indicate that 6-OHDA causes ROS-mediated DNA damage in a timeframe that precedes protein oxidation [7].

Ataxia telangiectasia-mutated kinase (Atm) and the Atm and Rad53-related kinase (Atr) are activated by phosphorylation in response to many DNA damage agents [33,34]. Therefore, we determined the phosphorylation state of Atm and Atr following treatment with 6-OHDA. Within 1 hour, 6-OHDA induced phosphorylation of Atr, but not Atm (Figure 7C).

Since *Puma *is a known transcriptional target of the p53 transcription factor and p53 is a target of ATR, we tested whether p53 was activated by 6-OHDA. Phosphorylation of p53 at Ser15 (p-p53) is known to be induced by DNA damage and leads to its accumulation and activation of transcriptional targets [35]. Western blot analysis demonstrated that 1 hour after 6-OHDA exposure, there is a significant increase in p-p53 levels, but not in overall p53 protein levels, in toxin-treated versus untreated cultures (Figure 7D, E). Immunocytochemistry revealed that p-p53 is localized primarily in the nucleus where it can activate transcription of its target genes (Figure 7F). Loss of Puma had no effect on phosphorylation of p53, which is consistent with a model that places p53 upstream of Puma (Figure 7D-F). Taken together, these data suggest that 6-OHDA-generated ROS rapidly activates the DNA damage repair pathway in dissociated DA cultures.

### p53-deficiency protects cell bodies and neurites against 6-OHDA

**Figure 8:**
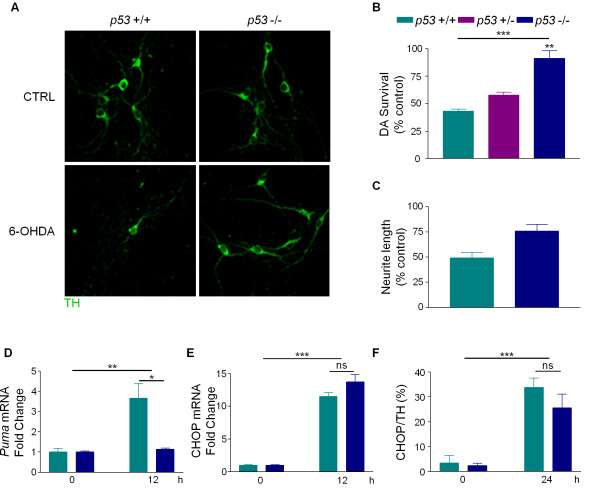
**p53 is required for 6-OHDA-induced cell death**. Primary cultures from individual pups from *p53 *+/- × *p53 *+/- matings were treated with 20 ¿M 6-OHDA. A) After 24 hours, cultures were fixed and stained for TH. B) TH-positive neurons were counted and survival was expressed as a percent of untreated control. Data were analyzed by one-way ANOVA (***, p < 0.001) with Bonferroni post-tests to compare to wild-type (-/-, **, p < 0.01). C) TH-positive neurite length was estimated using unbiased stereology and expressed as a percent of untreated control. Data were analyzed by Student's t-test (*, p < 0.05). D) Total RNA was isolated at the indicated times and *Puma *and *18S rRNA *RNA levels were analyzed by semi-quantitative RT-PCR. Data were analyzed by two-way ANOVA (treatment: **, p < 0.01; genotype: *, p < 0.05). E) Total RNA was isolated at the indicated times and *CHOP *and *GAPDH *RNA levels were analyzed by qPCR. Data were analyzed by two-way ANOVA (treatment: ***, p < 0.001; genotype: ns). F) Cells treated for 24 hours were co-stained for TH and CHOP. TH-positive and CHOP-positive cells were counted and the percentage of TH-positive cells that were also CHOP-positive was calculated. Data were analyzed by two-way ANOVA (treatment: ***, p < 0.001; genotype: ns). 11

To determine if p53 plays a critical role in 6-OHDA-induced cell death, dissociated dopaminergic cultures were prepared from *p53 *+/+, +/- and -/- littermates and treated with or without 6-OHDA for 24 hours prior to counting TH-positive neurons. In *p53 *+/+ cultures, 6-OHDA led to the loss of 50% of both TH-positive cell bodies and neurites; however, in *p53 *-/- cultures, only 15% of TH-positive cell bodies and 20% of TH-positive neurites were lost (Figure 8A-C). Non-dopaminergic cells in *p53 *-/- cultures were also protected against 6-OHDA, again confirming findings that 6-OHDA-induced cell death is less specific *in vitro *(data not shown) [23]. Thus, loss of *p53 *protects cell bodies and neurites against 6-OHDA and p53 is required for 6-OHDA-induced apoptosis.

Consistent with studies showing that *Puma *is a transcriptional target of p53, PUMA was not induced in *p53*-null cultures, but was significantly upregulated in *p53 *+/+ cultures (Figure 8D). Finally, to determine if the activation of p53 by 6-OHDA is distinct from the 6-OHDA-induced upregulation of UPR, CHOP levels were assessed in *p53 *+/+ and -/- cultures. Following 6-OHDA treatment, CHOP mRNA and protein levels were upregulated to similar levels in primary cultures derived from *p53 +/+ and -/- *mice (Figure 8E, F). Taken together, these data suggest that UPR is not downstream of p53 in this model and that the activation of p53 and upregulation of UPR represent two distinct responses to 6-OHDA.

## Discussion

**Figure 9:**
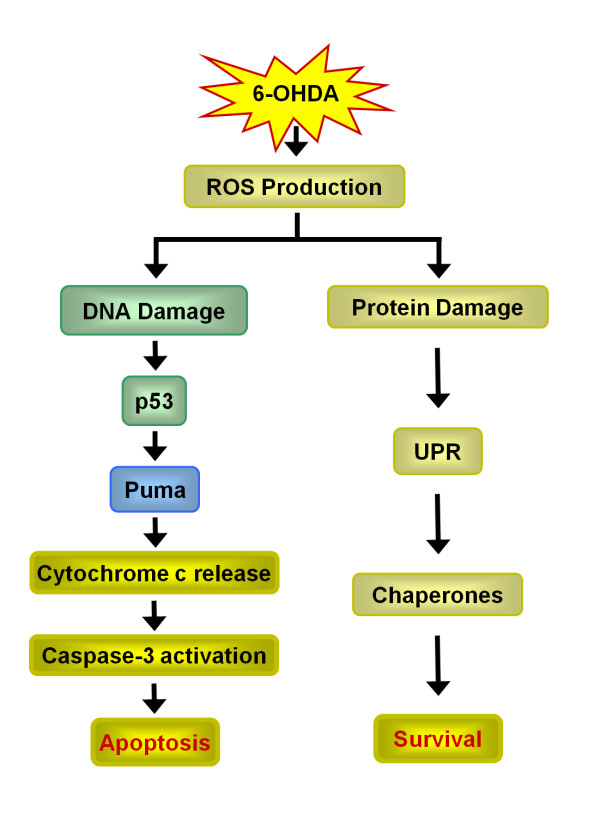
**Model of Puma action in response to 6-OHDA**. In this model, 6-OHDA-induced ROS production causes both DNA and protein damage. Oxidative protein damage activates UPR, leading to the upregulation of chaperones. DNA damage leads to the activation of the p53 DNA damage repair pathway and p53-mediated Puma upregulation leads to the induction of apoptosis.

Many PD-linked mutations are associated with protein aggregation and Lewy body formation, underscoring the role of aberrant protein handling in this disorder [36,37]. Although previous results established that 6-OHDA-triggered ER stress results in UPR, whether UPR and cell death are sequential or parallel events was not determined [7,27]. Here, we demonstrate that Puma is required for 6-OHDA-induced cell death both *in vitro *and *in vivo*. The loss of Puma blocks the induction of apoptosis but has no effect on the activation of UPR, suggesting that UPR and apoptosis are parallel events induced by 6-OHDA (Figure 9). In support of this, ATF3, a critical transcription factor involved in UPR, is not required for 6-OHDA-induced cell death. Instead, the DNA damage repair pathway is critical since loss of p53 or Puma prevents cell death during toxin treatment.

### Puma is required for 6-OHDA-induced cell death

We have demonstrated that Puma is required for 6-OHDA-induced caspase activation and cell death. In other systems, upregulation of Puma leads to cytochrome-c release and caspase activation [38,39]. Release of cytochrome-c from the mitochondria is an essential step to the activation of executioner caspases and apoptosis. Previously, we used subcellular fractionation and western blotting to demonstrate that 6-OHDA treatment leads to cytochrome-c redistribution and loss in primary cultures [7]. However, obtaining sufficient material for this method is not feasible when comparing two genotypes given the necessity of acquiring very large numbers of timed pregnant animals to isolate adequate amounts of mesencephalic tissue of each genotype. Alternatively, we sought to assess cytochrome-c release by immunocytochemistry. However, while loss of cytochrome-c staining reportedly represents cytochrome-c release in some systems [39], this was not true in dopaminergic neurons in primary culture since changes in staining did not mirror our subcellular fractionation and western blotting results (Additional File 1, Figure S1). Nevertheless, caspase-3 activation was not observed in *Puma *-/- neurons indicating that Puma is upstream of this executioner step in dopaminergic neurons (Figure 4).

### 6-OHDA-induced UPR does not mediate cell death

UPR is thought to activate both adaptive and apoptotic pathways, depending on the severity and duration of ER stress [22,40]. Pro- and anti-apoptotic proteins, such as Bcl-2, Bax and Bak, can also directly regulate UPR proteins, reinforcing the concept that UPR is a complex, highly regulated process intimately tied to cellular homeostasis as well as apoptosis [41]. Over the time course of 6-OHDA-triggered cell death, however, UPR appears to be primarily adaptive. The persistent activation of UPR even though the cells are protected from cell death suggests that UPR is not mediating cell death in this model. Conceivably, UPR might induce apoptosis in Puma deficient neurons over a longer time frame (2-3 days). However, in wild type cultures, UPR signalling pathways may still be protective while 6-OHDA induces p53/Puma-dependent cell death within 24 hours.

UPR is regulated by three interconnected pathways mediated by PERK, IRE1 and ATF6. The PERK arm of the UPR pathway triggers a cascade of transcription factors stemming from the phosphorylation of eIF2¿. ATF4 is immediately downstream, followed by ATF3 and then CHOP [42,43]. If PERK signalling were inducing cell death in dopaminergic neurons, then loss of ATF3 would have protected these cells from 6-OHDA. However, loss of ATF3 did not prevent 6-OHDA-mediated upregulation of Puma, caspase-3 activation or cell death even though, as expected, *ATF3*-deficiency reduced the upregulation of CHOP (Figure 6). Since loss of ATF3 reduced CHOP induction without affecting cell death or PUMA activation, this pathway is likely adaptive and distinct from the PUMA-cell death pathway.

The IRE1 and ATF6 branches of UPR also appear to be primarily adaptive in the 6-OHDA model. IRE1 is thought to activate the JNK pathway [44] and pharmacological inhibitors of JNK were ineffective against 6-OHDA (A. Bernstein and W. Holtz, unpublished observations) [27]. IRE1 can also regulate Bcl-2 family members [45]; however, neither the targeted over-expression of Bcl-2 in DA neurons nor Bax deficiency rescued cells from 6-OHDA-mediated apoptosis [46]. Although CHOP is also activated by ATF6, it is doubtful that this pathway is playing a role since temporally CHOP and Puma are induced at the same time point [7].

Finally, the loss of Puma had no effect on any of the UPR branches as splicing of Xbp-1 and the upregulation of *ATF3, BiP, CHOP, GADD34*,and *p58^IPK ^*occurred in both *Puma *+/+ and *Puma *-/- cultures (Figure 5). These data suggest that the entire UPR network is parallel to Puma. One caveat to this interpretation is a study demonstrating protection against 6-OHDA in *CHOP *knockout mice [47]. However, CHOP can be upregulated by a variety of cellular insults and may have been induced by distinct mechanisms owing in part to the chosen dosing paradigm. Moreover, the authors were unable to detect upregulation of other UPR markers [47].

### 6-OHDA induces cell death through the DNA damage repair pathway

Using DNA fragmentation assays or TUNEL staining, we previously demonstrated single strand DNA breaks 18-24 hours after 6-OHDA treatment suggesting DNA oxidation was a late event (Holtz, Kim-Han, and O'Malley, unpublished observations) [48]. However, these methodologies lack the sensitivity to measure rapidly occurring changes in DNA structure. In contrast, antibodies directed against PAR, phosphorylated Atr and p53 revealed very rapid changes to the DNA damage repair pathway (Figure 7). These findings are consistent with results from other cell systems demonstrating that p53 is required for 6-OHDA-induced cell death (Figs. 7,8) [49,50]. Loss of p53 also prevents the upregulation of *Puma*, indicating that p53 is upstream of *Puma *in this system (Figure 9). In contrast, the UPR marker, CHOP, is unaffected by the loss of p53, providing further evidence that 6-OHDA-induced apoptosis and UPR represent parallel processes. These findings are in agreement with previous results demonstrating the activation of p53 by 6-OHDA and protection by inhibitors of p53 against 6-OHDA [49,50].

### Cell body loss and neurite loss are separate processes in dissociated DA neuron

New data suggest that axonal impairment may play an early, critical role in PD. Hence, loss of neurites and cell bodies may occur by molecularly distinct programs [24]. Results from the dissociated midbrain cultures support this notion as loss of *p53 *protects both cell bodies and neurites, whereas loss of PUMA protects only cell bodies (Figure 2, data not shown). This suggests that the pathway splits downstream of p53. PUMA mediates death of the cell body and a separate p53-dependent pathway is responsible for loss of neurites. In vivo, however, loss of Puma protected both the SN cell bodies and DA innervation of the striatum (Figure 3). Additional studies are underway to understand these differences.

## Conclusions

Taken together, the results of the present study suggest that the activation of UPR does not mediate cell death but may represent a protective mechanism employed by dopaminergic neurons in response to 6-OHDA-induced protein damage. Instead, DNA damage may lead to activation of a p53- and Puma-dependent apoptotic cascade. There is a large body of evidence supporting both the activation of UPR pathways and p53-dependent pathways in toxin models of PD and PD itself. Therefore, elucidating which of these mechanisms is protective and which leads to cell death will help in developing better interventions for PD.

## Methods

### Animals

Animals were treated in accordance with the National Institutes of Health *Guide for the Care and Use of Laboratory Animals*. Wild-type C57/Bl6 mice were from Charles River Laboratories (Wilmington, MA). *Puma *knockout mice were previously generated and characterized [16]. *ATF3 *knockout mice were generated and provided by Dr. Tsonwin Hai (Ohio State University) [29]. *p53 *knockout mice were provided by Dr. Helen Piwnica-Worms (Washington University Medical School) [51].

### 6-OHDA injections

Injections of 10 ¿g 6-OHDA were done as described using the coordinates A 1.0, L 2.5 mm [52]. 6-OHDA was dissolved in N_2_-bubbled water at a concentration of 5 ¿g/¿l and injected at a rate of 0.5 ¿l/min for 4 m. Mice were sacrificed one month after injection and brains were removed for analysis. Preliminary experiments demonstrated that this dose produced ~50% loss of dopaminergic neurons in the SN 1 month after injection.

### Tissue preparation

After removal of the brain, striata were dissected and the remaining brain was fixed and cryoprotected as previously described [53]. Striata were immediately homogenized for high-pressure liquid chromatography (HPLC) [53]. Coronal sections (50 ¿M) spanning the SN (A - 2.8 to -3.8) were cut on a microtome for immunocytochemistry.

### Immunohistochemistry and stereology

Every fourth SN section was stained with sheep polyclonal anti-TH antibody (1:2000, Novus Biologicals, Littleton, CO) followed by Alexa 488 conjugation secondary (1:1000, Molecular Probes, Eugene, OR). Sections were mounted and coverslipped with Vectashield with DAPI (Vector Labs, Burlingame, CA). StereoInvestigator software (MicroBrightField, Williston, VT) was used to perform unbiased stereological counts of TH immunoreactive cell bodies in the SNpc using the optical fractionator method [25]. Counting was performed with a 63x oil objective. The estimated total number of TH neurons was calculated based on the following formula: N = Q^- ^×1/ssf × 1/asf × t/h, where N is the estimate of the total number of cells, Q^- ^is the number of objects counted, ssf is the section sampling fraction, asf is the area sampling fraction, and t/h is the actual section thickness divided by the height of the dissector. Gundersen (m = 1) coefficients of error were less than 0.1. The number of cells on the side ipsilateral to the injection was compared to the number of cells on the contralateral side by calculating the ipsilateral to contralateral ratio. For imaging, sections were stained with mouse monoclonal anti-TH antibody (1:5000, Millipore, Billerica, MA) with DAB detection (Vector Labs). Sections were mounted and coverslipped with Vectashield (Vector Labs). Images were acquired on a Nikon 90i with Volocity software (Improvision, Waltham, MA).

### Dopamine measurements

Striatal lysates were processed as described [53], diluted in MD-TM mobile phase (ESA, Chelmsford, MA) and separated on an ESA MD-150 column with a Coulochem III and EZChromElite software (ESA). Samples were analyzed in triplicate; ipsilateral to contralateral ratios were calculated.

### Cell culture

Embryonic day 14 (E14) CF1 murine midbrains (Charles River Laboratories, Wilmington, MA, USA) were prepared as described [6]. To assess survival of neurons after 6-OHDA treatment, mice heterozygous for *Puma *or *p53 *were mated to produce wild type, heterozygous, and homozygous deficient embryos. Cultures were derived from individual pups and pups were individually genotyped. Comparisons were performed between littermates. Due to the limited amount of tissue harvested from individual pups, all other experiments were performed by mating homozygous knockout animals or wild-type animals and pooling tissue from all pups in each litter. After seven days *in vitro*, cells were treated with 20 ¿M 6-OHDA, a dose that produces 50% loss of dopaminergic neurons 24 hours after treatment.

### Reverse Transcription PCR

Dissociated midbrain neurons were plated in 12-well plates, treated with 6-OHDA and/or pretreated with N-acetyl cysteine (NAC; Sigma). Cultures were washed with PBS, total RNA was extracted (RNeasy Mini Kit; Qiagen, Valencia, CA) and then reverse transcribed (High Capacity cDNA Reverse Transcription Kit; Applied Biosystems, Foster City, CA). Levels of *Puma*, *Xbp-1 *and *18S rRNA *were analyzed by semi-quantitative reverse transcription PCR (RT-PCR) using primers specific for the gene of interest. PCR products were resolved with polyacrylamide gel electrophoresis, visualized (SYBR Safe DNA; Invitrogen) and image (Storm PhosphorImager; Molecular Dynamics, Piscataway, NJ). Band intensities were measured (ImageQuant; Amersham Biosciences, Piscataway, NJ) and *Puma *and *Xbp-1 *levels were normalized to 18S rRNA levels and then compared to levels in untreated samples. Levels of *ATF3, CHOP, BiP, GADD3, p58^IPK ^*and *GAPDH *were determined by quantitative real-time PCR (qPCR). cDNAs were amplified with Power SYBR Green PCR Master Mix (Applied Biosystems) using gene-specific primers (Table 1). Detection was performed with the Applied Biosystems Prism 7000. All PCR reactions were done in triplicate. All genes were normalized to GAPDH and compared to untreated samples.

**Table 1:** Primers for Reverse Transcription PCR

Gene	Primer 1	Primer 2
*18s rRNA*	5'-GGGAACGCGTGCATTTATCAG-3',	5'-CGCTATTGGAGCTGGAATTAC-3'

*ATF3*	5'-TGCCAAGTGTCGAAACAAGAA-3'	5'-CGGTGCAGGTTGAGCATGTA-3'

*BiP*	5'-CTTCAATGATGCCCAGCGA-3'	5'-CCAGGCCATATGCAATAGCAG-3'

*CHOP*	5'-TATCTCATCCCCAGGAAACG-3'	5'-GATGTGCGTGTGACCTCTGT-3'

*GADD34*	5'-GAGATTCCTCTAAAAGCTCGG-3'	5'-CAGGGACCTGGACGGGCAGC-3'

*GAPDH*	5'-TGCCCCCATGTTTGTGATG-3'	5'-TGTGGTCATGAGCCCTTCC-3'

*p58^IPK^*	5'-TCCTGGTGGACCTGCAGTACG-3'	5'-CTGCGAGTAATTTCTTCCCC-3'

*Puma*	5'-ACGACCTCAACGCGCAGTA-3'	5'-CTAGTTGGGCTCCATTTCTGG-3'

*Xbp-1*	5'-TAGAAAGAAAGCCCGGATGA-3'	5'-CTCTGGGGAAGGACATTTGA-3'

### Western blotting

Dissociated midbrain neurons were plated in 48-well plates and treated as described above for RT-PCR experiments. Lysates were collected and prepared as described [7]. Rabbit polyclonal antibodies against p-p53 (Ser15), p-ATR (Ser428), p-ATM (Ser1981), p-eIF2¿ (Ser51), cleaved caspase-3 and HRP-linked anti-rabbit antibodies were from Cell Signaling Technologies (Beverly, MA). Rabbit polyclonal anti-ATF3 and mouse monoclonal anti-CHOP/Gadd153 were from Santa Cruz Biotechnologies (Santa Cruz, CA). Rabbit polyclonal anti-Puma, mouse anti-actin and HRP-linked anti-mouse antibody were from Sigma (Saint Louis, MO). Mouse monoclonal anti-p53 was from EMD Biosciences (San Diego, CA). Specific protein bands were detected and analyzed by chemiluminescence substrate detection and quantitative fluoroimaging as previously described [7].

### Immunocytochemistry

Dissociated midbrain neurons were plated and fixed as described [6]. Cultures were stained with sheep polyclonal anti-tyrosine hydroxylase (TH) (Novus Biologicals, Littleton, CO) and Alexa488 ¿-sheep (Molecular Probes, Carlsbad, CA). Cultures were co-stained for various markers using the following antibodies: mouse monoclonal ¿-NeuN (Chemicon, Billerica, MA), cleaved caspase-3, mouse monoclonal ¿-cytochrome-c (Promega, Madison, WI), mouse monoclonal ¿-CHOP (Santa Cruz), ¿-p-p53 or mouse monoclonal ¿-Poly (ADP-Ribose) (PAR; Alexis Biochemicals, San Diego, CA). Cy3-conjugated secondary antibodies were purchased from Jackson Labs (Bar Harbor, ME). In all cases cells were counted using unbiased stereological methods (Stereo Investigator, MicroBrightField, Williston, VT). The estimated total number of TH neurons in the culture dish was calculated based on the following formula: N = Q^- ^×1/ssf × 1/asf, where N is the estimate of the total number of cells, Q^- ^is the number of objects counted, ssf is the section sampling fraction and asf is the area sampling fraction. Gundersen (m = 1) coefficients of error were less than 0.1. TH-positive neurite length was estimated by an unbiased stereological method (Petrimetrics, Stereo Investigator). Images were acquired by confocal microscopy (Olympus Fluoview 500, Olympus, Center Valley, PA) and processed in ImageJ.

### Statistical analysis

GraphPad Prism software (San Diego, CA) was used for statistical analysis. All data were collected from a minimum of three independent experiments. The significance of effects between control and drug treatment conditions was determined by Student's t-test or one-way ANOVA with Bonferroni Multiple Comparisons tests. The significance of effects between genotypes and drug treatment conditions was determined by two-way ANOVA with Bonferroni post-tests.

## Competing interests

The authors declare that they have no competing interests.

## Authors' contributions

AB participated in experimental design, carried out all experiments described except for western blotting for Puma and drafted the manuscript. SG performed western blots for Puma. GZ provided the Puma -/- mice. KO was involved in the design of experiments and production of the manuscript. All authors participated in revising and editing the final manuscript.

## Supplementary Material

Additional file 1 **Figure S1: Loss of cytochrome-c detected by immunocytochemistry does not parallel redistribution of cytochrome-c detected by fractionation and western blotting**. Cultures derived from *Puma *+/+ and -/- animals were treated with 20 ¿M 6-OHDA for 18 h. A) Cells were fixed and stained for TH and cytochrome-c. B) Images were analyzed in ImageJ to determine the area of cytochrome-c staining over threshold in TH-positive neurons. Data was analyzed by two-way ANOVA (treatment: ***, p < 0.001; genotype: ns). Click here for file
