# Supplementary figures and images for: 6-OHDA generated ROS induces DNA damage and p53- and PUMA-dependent cell death
Source: Mol Neurodegener. 2011 Jan 6;6:2. doi: 10.1186/1750-1326-6-2 (PMC3025875; doi:10.1186/1750-1326-6-2)

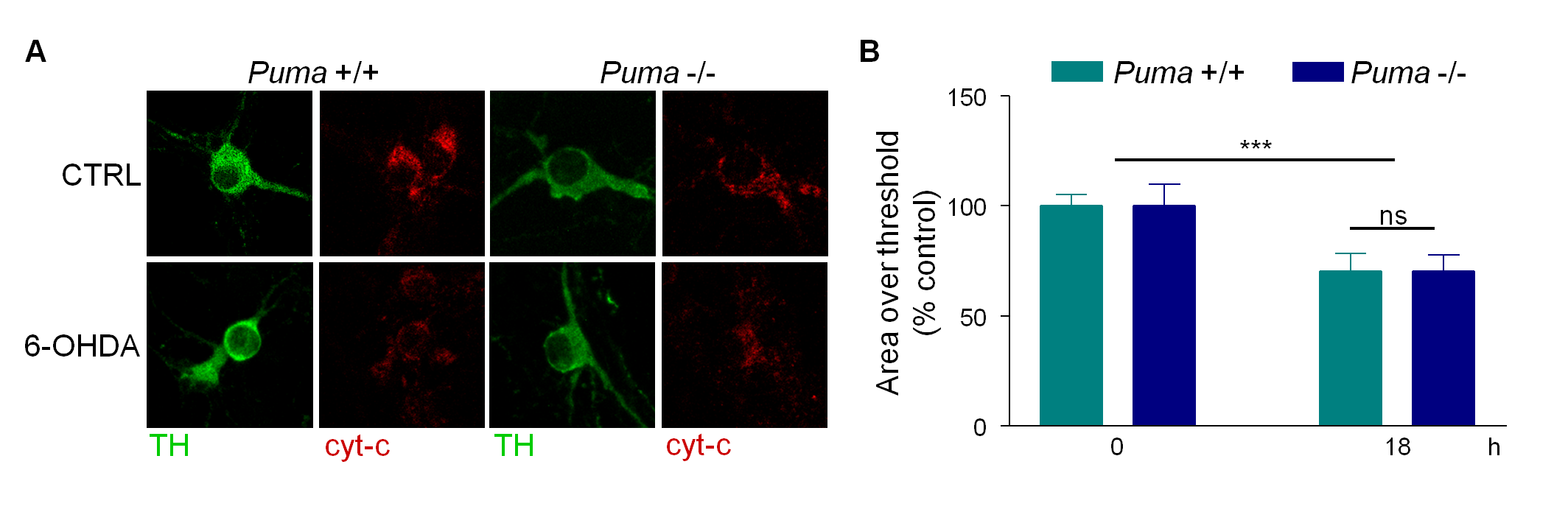

Supplement: Additional file 1 — Figure S1: Loss of cytochrome-c detected by immunocytochemistry does not parallel redistribution of cytochrome-c detected by fractionation and western blotting. Cultures derived from Puma +/+ and -/- animals were treated with 20 ¿M 6-OHDA for 18 h. A) Cells were fixed and stained for TH and cytochrome-c. B) Images were analyzed in ImageJ to determine the area of cytochrome-c staining over threshold in TH-positive neurons. Data was analyzed by two-way ANOVA (treatment: ***, p < 0.001; genotype: ns). [file 1750-1326-6-2-S1.TIFF]
